# Interactive shape and color representation in visual working memory for colored objects in the human occipitotemporal cortex

**DOI:** 10.1162/IMAG.a.1049

**Published:** 2025-12-05

**Authors:** Yaoda Xu, Benjamin Swinchoski, JohnMark Taylor, Marvin Chun

**Affiliations:** Yale University, New Haven, CT, United States; Columbia University, New York, NY, United States

**Keywords:** visual working memory, visual short-term memory, visual object representation, posterior parietal cortex, occipitotemporal cortex, fMRI, vision

## Abstract

Two defining visual features of a real-world object are its shape and color. While previous fMRI pattern decoding studies showed that these features are represented in a largely orthogonal and independent manner in the human occipitotemporal cortex (OTC) during visual perception, how they are coded together in visual working memory (VWM) remains unknown. Here, we asked human participants to retain a real-world object’s shape and color together in VWM. Using fMRI pattern decoding, we trained a linear classifier to decode two object shapes in one color to then decode the same two object shapes either in the same color (within-decoding) or in a different color (cross-decoding). During VWM encoding, replicating prior findings, we found significant cross-decoding and no drop in cross-decoding compared to within-decoding across OTC, indicating orthogonal and independent shape and color coding. During VWM delay, we again found significant cross-decoding, but more critically, a significant cross-decoding drop in OTC shape-selective areas and ventral color areas, indicating interactive shape and color coding in these areas in VWM. To our knowledge, this is the first time that interactive shape and color representations are reported in VWM in the human brain using fMRI pattern decoding. Sensory representations of an object’s shape and color are thus transformed during VWM delay, likely tracking the demand of VWM to enable more efficient information storage.

## 1 Introduction

Shape and color are two salient visual features that enable us to differentiate objects in our everyday visual environment. In this study, we examined how the coding of shape and color may change between perception and visual working memory (VWM).

### 1.1 The coding of shape and color in visual perception

By measuring fMRI response amplitude differences, prior research has identified distinctive regions in the human higher visual cortex selective for object shapes and colors during visual perception. Specifically, regions in the lateral occipitotemporal (LOT) and ventral occipitotemporal (VOT) cortex show a preference for intact over scrambled object shapes (Grill-Spector et al., 1998; Kourtzi & Kanwisher, 2000; Malach et al., 1995; Orban et al., 2004), and several ventral color regions show a preference for colored over greyscaled images (Lafer-Sousa et al., 2016). While these univariate results suggest that shapes and colors might be exclusively or predominantly processed in these localized regions, using fMRI pattern analysis, we recently reported successful decoding for both color and shape in a perceptual task throughout the ventral and lateral human visual cortex, including visual areas V1 to V4, and color- and shape-selective regions in the ventral and lateral occipitotemporal cortex (OTC) (Taylor & Xu, 2022). Although there was a decoding bias in shape- and color-selective regions toward their univariate preferred feature (i.e., with decoding performance being higher for the preferred than the non-preferred feature), the ubiquity of successful shape and color decoding throughout the ventral and lateral visual cortex shows their broader representation across the visual processing hierarchy. This is consistent with results from prior neurophysiological studies (for a brief review of this literature, see Rentzeperis et al., 2014; Taylor & Xu, 2022).

In additional analyses, we found that shape and color are coded in a largely independent manner across ventral and lateral visual regions in the human brain. This is especially true for complex rather than for simple shapes, such that a linear pattern classifier trained to decode two shapes in one color can generalize its decoding performance to the same two shapes in a different color, with no significant drop in cross-decoding performance (Taylor & Xu, 2022). Overall, shape and color are coded predominantly in a largely orthogonal and independent manner in the visual representational space throughout the visual processing hierarchy, with changes in the value of one feature having a minimal impact on the representation of the other. This is unlike what has been found in convolutional neural networks (CNNs) trained for object recognition, which show interactive coding of shape and color at higher levels of visual processing, indicating a discrepancy in how these two types of features are coded in the human brain and CNNs (Taylor & Xu, 2021). The early separation of shape and color in the human brain also contrasts with the separation of shape from other features such as position, size, or the spatial frequency content of an image, which only occurs during later stages of visual processing (Xu & Vaziri-Pashkam, 2022).

### 1.2 Information representation in visual working memory

In everyday vision, we often need to maintain visual input for a short time in VWM when it is no longer in view. Over the last decade and a half, fMRI pattern decoding studies have shown that the content of VWM can be successfully decoded in sensory areas, such as early visual areas (e.g., Bettencourt & Xu, 2016a; Harrison & Tong, 2009; Serences et al., 2009). While earlier studies have argued that VWM representations are copies or extensions of perceptual representations (e.g., D’Esposito and Postle, 2015; Serences et al., 2009), more recent work has reported substantial transformations between perceptual and VWM representations (e.g., Duan & Curtis, 2024; Kwak & Curtis, 2022; Li & Curtis, 2023; Xu, 2023, 2025; Yan et al., 2023; see Kiyonaga & Serences, in press, for an extended review). This is likely due to information maintained in VWM in early visual areas being driven largely by feedback from regions in the prefrontal cortex (PFC) and posterior parietal cortex (PPC) (e.g., Lawrence et al., 2018; Liebe et al., 2012; van Kerkoerle et al., 2017). Such feedback not only can reestablish the VWM signal in OTC after it was disrupted by distraction (van Kerkoerle et al., 2017) but also can alter the format and the content of VWM representations in OTC (e.g., Xu, 2023). These transformed VWM representations in sensory areas have been shown to better meet the demands of VWM tasks, such as forming more streamlined representations (Kwak & Curtis, 2022), mediating behavior (Li & Curtis, 2023), and tracking the task-relevant representations in PPC (Xu, 2023). This raises an intriguing question: given that shape and color are represented in an orthogonal and independent manner during visual perception in OTC, do their representations maintain independence during VWM?

It is possible that object shapes and colors are coded in an orthogonal manner in VWM as an extension of their perceptual representation, consistent with the sensory account of VWM. However, rather than sustaining two independent representations, when the task requires the retention of both features of an object, given the limited VWM storage capacity, VWM may maintain a partially integrated representation to reduce the overall storage load and more effectively retain both object features at the same time. This could protect them from interference and decay during VWM delay and predict interactive coding of object shapes and colors in VWM.

### 1.3 The present study

To test whether color and shape representations remain independent or become integrated beyond initial perceptual encoding, in this study, we asked human participants to retain a real-world object’s shape and color together in VWM. As was done previously to measure interactive feature coding (Taylor & Xu, 2022, 2024; Xu, 2024), using fMRI pattern decoding, we used a linear classifier trained to decode a pair of object shapes in one color to decode the same pair of objects either in the same color (within-decoding) or in a different color (cross-decoding). During VWM encoding, replicating Taylor and Xu (2022), we expect to see no cross-decoding drop, indicating orthogonal and independent shape and color coding during initial perception. During VWM delay, however, if the two features are coded in an integrated manner, we expect to see a significant cross-decoding drop. If the orthogonal shape and color representational scheme found in perception extends to VWM maintenance, we should find no cross-decoding drop.

## 2 Materials and Methods

### 2.1 Participants

Twelve healthy human participants (six females) with normal or corrected to normal visual acuity and normal color vision, all right-handed and aged between 18–35, took part in the study. All participants gave their informed consent prior to the study and received payment for participation. The study was approved by the Committee on the Use of Human Subjects at Yale University. This sample size was determined based on prior work (e.g., N = 11 for Kwak & Curtis, 2022; and N = 14, Li & Curtis, 2023; Ns = 12 and 13 for the two experiments in Taylor & Xu, 2022; N = 13 in Taylor & Xu, 2024; N = 12 in Xu, 2023; N = 14 in Xu, 2024; and N = 12 in Yu & Shim, 2017).

### 2.2 Main VWM experiment

**Fig. 1.**
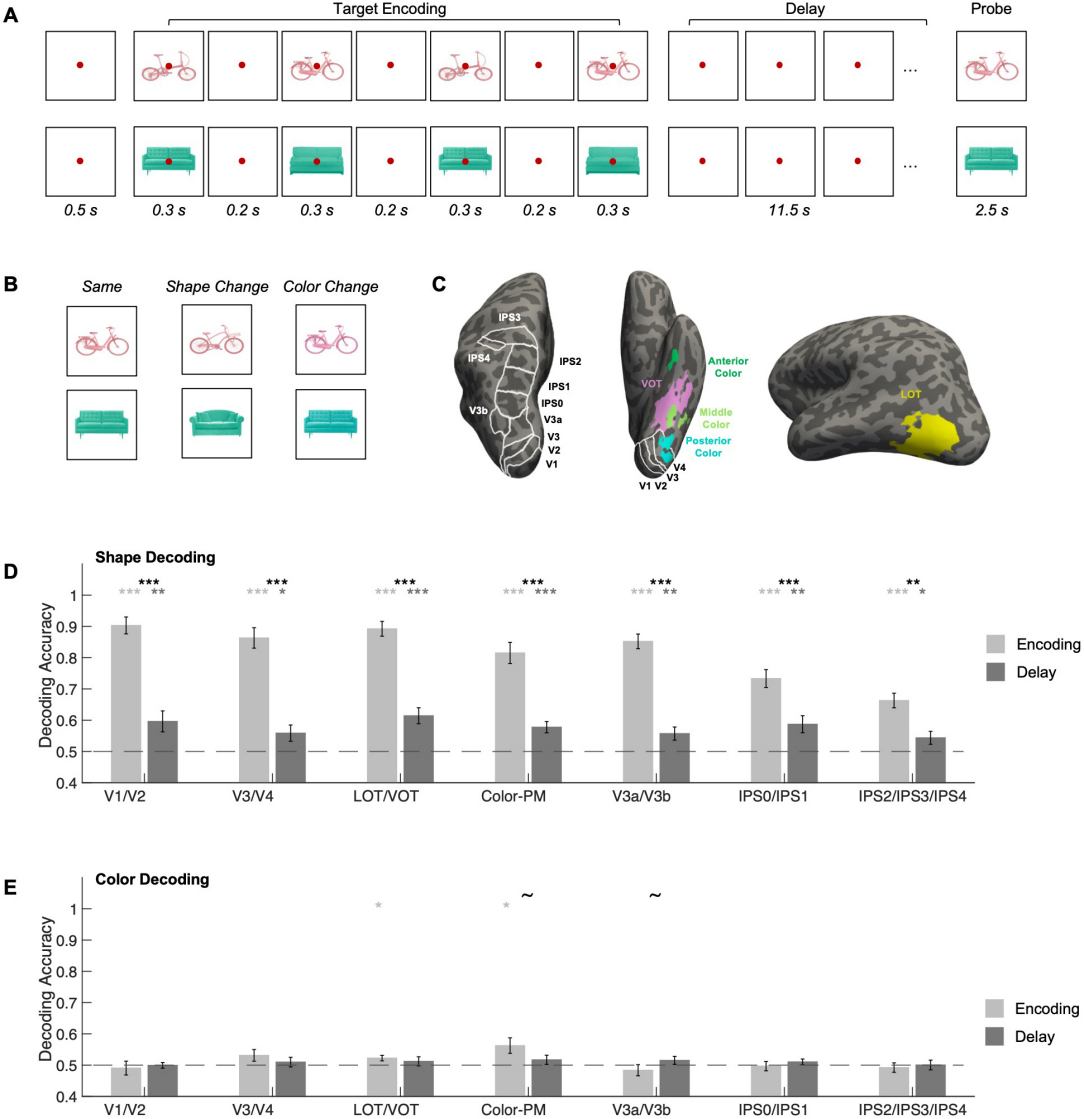
Decoding illustration, experimental design, example brain regions of interest (ROIs), and within-feature decoding results. (A) Example trials showing the trial sequence. In each trial, two exemplars of the same object in the same color were shown sequentially twice during the encoding period. After an extended blank delay period, a probe is shown. (B) Example probe objects. In “Same” trials, the probe object would be one of the target objects; in “Shape Change” trials, the probe object would be another exemplar of the same object in the same target color; and in “Color Change” trials, the probe object would be one of the target objects appear in another shade of the same color. The entire object exemplar and color set is shown in Supplementary Figure S1. (C) Example ROIs shown on the inflated cortical surfaces. Note that both V4 and VOT have some overlap with the color areas. These overlaps were taken into consideration in our analysis (see Section 3). (D) Object shape decoding accuracy during VWM encoding and delay. (E) Object color decoding accuracy during VWM encoding and delay. The light grey and medium grey symbols above the bars mark the decoding significance of each bar compared to chance (.5). The black symbols mark the significance of the difference between each bar pair. Error bars indicate s.e. ~*p* < .1, *p < .05, **p < .01, ***p < .001.

This experiment used two types of objects sharing roughly the same outline (bikes and couches), appearing in two colors (red and green) (Fig. 1A). The objects used here have been used in several of our prior studies examining object, attention, and VWM representations (Jeong & Xu, 2017; Mocz et al., 2023; Xu & Jeong, 2015; Xu, 2024). Each VWM trial contained a sequential central presentation of two exemplars from the same object in the same exact color (Fig. 1A). Based on the results from several pilot studies, this was done to increase task difficulty, ensure that a visual code was employed to retain VWM representation, and increase object shape decoding accuracy. As shown in Figure 1B, the probe object at the end of the VWM delay was either one of the target exemplars in the exact same color (no change), one of the target exemplars in a different shade of the target color (color change), or another exemplar of the same object in the exact same target color (shape change). Exemplars of the same objects were shown at the same vantage point (i.e., front view for couches and side view for bikes) to increase the similarity between the exemplars of the same object (see Supplementary Fig. S1 for the entire set of object exemplars and colors used). To colorize the stimuli, images were converted to the CIELUV color space and had their hue uniformly set to one of six different shades of red or six different shades of green. Each set of six hues was evenly spaced, with the red and green hues on opposite sides of the color wheel (i.e., 180° apart). Images were then tuned such that the mean and standard deviation of the luminance (not including the background) were the same across all stimuli, and the saturation (defined as $\sqrt{u^{2}+v^{2}},\;$ where u and v are the two chromaticity coordinates in the CIELUV color space) was the same uniform value across all objects.

All images were placed on a white square (subtending 9.73° × 9.73°) and shown on a larger grey background. A given trial contained the presentation of the two target exemplars, a prolonged blank delay, and a probe object (Fig. 1A). To ensure that participants successfully encoded the two target exemplars, these target exemplars were shown sequentially twice. Each trial was 16 s long, with the timing of the different events as follows: fixation (.5 s; in the form of a looming red dot to alert the participant to the imminent presentation of the target images), first target exemplar (.3 s), blank period (.2 s), second target exemplar (.3 s), blank period (.2 s), first target exemplar (.3 s), blank period (.2 s), second target exemplar (.3 s), extended delay period with a red fixation dot (11.3 s), and probe image (2.4 s). Participants pressed the left button on the two-button button box with their right index finger if the probe was an exact exemplar and shade of color as one of the target objects, and the right button on the button box with their right middle finger if either the exemplar or the shade of color changed. Each run started and ended with an 8-s blank period with a blue fixation dot and contained 16 trials, four for each of the four object and color combinations (2 objects x 2 colors). Successive VWM trials were sandwiched by a blank period with a blue fixation dot. Of the 15 such inter-trial blank periods, three were 8 s long, and 12 were 2.4 s long, and they were randomly distributed. Twelve runs of data were collected from each participant, with each run lasting 5 min 12 s.

The design of the present study was modeled closely after Harrison and Tong (2009), who asked participants to remember different exemplars from two orientation categories (i.e., with exemplars varying +/-3 or +/-6 degrees from 25 or 115 degrees). Responses in Harrison and Tong (2009) were averaged over exemplars within each orientation category, and decoding was performed at the category level (25 vs. 115 degrees). Following this design, in the present study, target decoding was performed at the colored object level (e.g., red bikes vs. red couches) by including all trials containing exemplars from the same object in different shades of the same color (e.g., all trials with bike exemplars in different shades of red were treated as red bike trials).

### 2.3 Localizer experiments

#### 2.3.1 Topographic visual regions

These regions were mapped with flashing checkerboards using standard techniques (Sereno et al., 1995; Swisher et al., 2007) with parameters optimized following Swisher et al. (2007) to reveal maps in the parietal cortex. Specifically, a polar angle wedge with an arc of 72° swept across the entire screen (19.07 × 13.54° of visual angle). The wedge had a sweep period of 32 s, flashed at 4 Hz, and swept for 8 cycles in each run (for more details, see Swisher et al., 2007). Participants completed 4 to 6 runs, each lasting 4 min 36 s. All participants were asked to detect a dimming that could occur anywhere within the polar angle wedge, thereby ensuring attention to the whole wedge.

#### 2.3.2 Lateral and ventral occipitotemporal regions (LOT and VOT)

To identify these ROIs, following Kourtzi and Kanwisher (2000) and as we have done previously (Jeong & Xu, 2017; Vaziri-Pashkam & Xu, 2017, 2019; Vaziri-Pashkam et al., 2019; Xu & Vaziri-Pashkam, 2019; Taylor & Xu, 2022), participants viewed blocks of objects and scrambled objects (all subtended approximately 9.73° × 9.73°). The images were photographs of greyscale common objects (e.g., cars, tools, and chairs) and phase-scrambled versions of these objects. Participants monitored a slight spatial jitter, which occurred randomly once in every 10 images. Each run contained 4 blocks each of the objects, phase-scrambled objects, and two other conditions that were used to define another brain region. Each block lasted 16 s and contained 20 unique images, with each appearing for 750 ms and followed by a 50-ms blank display. Besides the stimulus blocks, 8-s fixation blocks were included at the beginning, middle, and end of each run. Each participant was tested with 2 runs, each lasting 4 min 40 s.

#### 2.3.3 Posterior, middle, and anterior color patches

To localize the color-selective regions in the ventral temporal cortex, two runs of a color localizer were conducted. Following Lafer-Sousa et al. (2016) and as in Taylor and Xu (2022), participants viewed 16-s blocks consisting of either colorful, highly saturated natural scene images selected from the online Places scene database (Zhou et al., 2018) or greyscaled versions of these images. Participants responded when an image jittered back and forth, which occurred twice per block. Images subtended 9.73° of visual angle and were each presented for 750 ms, with a 50-ms blank period between successive stimulus presentations within a block. Each run contained 16 blocks, 8 for each of the two stimulus types, for a total run duration of 4 min 32 s, including an initial 20-s fixation block and an 8-s fixation block in the middle and at the end of the run.

### 2.4 MRI method

Each participant completed one experimental session (1.5 h) and one localizer session (1.5 h) containing topographic mapping and functional localizers. MRI data were collected using a Siemens Prisma 3T scanner, with a 32-channel receiver array head coil. Participants lay on their backs inside the scanner and viewed the back-projected display through an angled mirror mounted inside the headcoil. The display was projected using an LCD projector at a refresh rate of 60 Hz and a spatial resolution of 1280 × 1024. An Apple MacBook Pro laptop was used to create the stimuli and collect the motor responses. Stimuli were created using MATLAB and Psychtoolbox (Brainard, 1997).

A high-resolution T1-weighted structural image (0.8 × 0.8 × 0.8 mm) was obtained from each participant for surface reconstruction. All blood-oxygen-level-dependent (BOLD) data were collected via a T2*-weighted echo-planar imaging pulse sequence that employed multiband radiofrequency (RF) pulses and simultaneous multi-slice (SMS) acquisition. For both the main experiment and the localizers, 72 axial slices (2 mm isotropic), 0 skip, covering the entire brain, were collected (time to repeat (TR) = 800 ms, time to echo (TE) = 37 ms, flip angle = 52°, SMS factor = 8).

### 2.5 Data analyses

FMRI data were analyzed using FreeSurfer (surfer.nmr.mgh.harvard.edu), FsFast (Dale et al., 1999), and in-house MATLAB codes. LibSVM software (Chang & Lin, 2011) was used for the MVPA support vector machine (SVM) analysis. FMRI data preprocessing included 3D motion correction and linear and quadratic trend removal. After reconstructing the inflated 3D cortical surface of each participant using the high-resolution anatomical data, we projected the fMRI data from that participant onto their native cortical surface. As was done in recent studies (Xu, 2023, 2024), all fMRI responses were analyzed directly on the inflated cortical surface (vertices) rather than on the cortical volume (voxels) of each participant, including ROI definition and the main VWM analysis, as surface-based analysis has been shown to exhibit more sensitivity and better spatial selectivity (Brodoehl et al., 2020; Oosterhof et al., 2011).

#### 2.5.1 ROI definitions

Following the detailed procedures described in Swisher et al. (2007) and as was done in our prior publications (Bettencourt & Xu, 2013, 2016a, 2016b; Vaziri-Pashkam & Xu, 2017, 2019; Vaziri-Pashkam et al., 2019; Xu & Vaziri-Pashkam, 2019), by examining phase reversals in the polar angle maps, we identified areas V1 to V4, V3a, V3b, and IPS0 to IPS4 in each participant (Fig. 1C). Following Kourtzi and Kanwisher (2000) and as was done in our prior studies (Jeong & Xu, 2017; Taylor & Xu, 2022, 2024; Vaziri-Pashkam & Xu, 2017, 2019; Vaziri-Pashkam et al., 2019; Xu & Vaziri-Pashkam, 2019), LOT and VOT were then defined as clusters of continuous voxels in the lateral and ventral occipital cortex, respectively, that responded more to the intact than to the scrambled object images (Fig. 1C). LOT and VOT loosely correspond to the location of the lateral occipital (LO) cortex and posterior fusiform gyrus (pFs) (Grill-Spector et al., 1998; Kourtzi & Kanwisher, 2000; Malach et al., 1995) but extend further into the temporal cortex in our effort to capture the continuous activations often seen extending into the ventral temporal cortex. Following Lafer-Sousa et al. (2016) and Taylor and Xu (2022), posterior, middle, and anterior color regions were identified in the ventral temporal cortex as clusters of voxels responding more to colored images than to greyscaled versions of the same images (Fig. 1C). While we were able to identify posterior and middle color regions in every hemisphere of every participant, as in prior studies (Lafer-Sousa et al., 2016; Taylor & Xu, 2022), we were unable to localize the anterior color regions consistently in each hemisphere across participants. This was likely due to this color region’s location being close to the ear canals, where large MRI susceptibility effects and signal dropoff could occur. Consequently, we only included posterior and middle color regions in our analysis. The same ROIs from the left and right hemispheres were combined to form a single bilateral ROI, as was done in prior VWM studies (e.g., Bettencourt & Xu, 2016a; Harrison & Tong, 2009; Li & Curtis, 2023; Serences et al., 2009; Xu, 2023 & 2024). The average number of vertices obtained from each ROI across the participants is reported in Supplementary Figure S2A.

As shown in Figure 1C and noted in Taylor and Xu (2022), both V4 and VOT had some overlap with the color areas, with on average 49% of V4 and 31% of VOT vertices in the color areas. This overlap was taken into consideration in our data analysis (see later).

#### 2.5.2 VWM decoding analysis

With the length of our VWM trials being 16 s, for each surface vertex, we estimated the fMRI response amplitude at each TR from the onset of the trial up to 32 s, totaling 40 TRs (with each TR being 800 ms). This was done separately for the trials in each of the four conditions. To obtain these estimates, we first constructed 40 finite impulse response (FIR) functions corresponding to each TR of each condition’s trials. Due to each condition appearing only four times in a run, the short intertrial interval (mostly 2 s), and the lag in hemodynamic responses, applying a general linear model (GLM) to each individual run would likely be an underpowered approach. To increase power and obtain more reliable amplitude estimates for the FIRs, we followed the procedure developed in recent studies (Xu, 2023, 2024) and used a split-half approach to combine two runs together, as detailed below, before applying GLM modeling to derive the beta weight estimate for each of the 160 FIR functions (40 TRs x 4 categories). To obtain independent training and testing data for pattern decoding, we split the runs into odd and even halves, with each split containing six runs. We then applied a GLM to each 2-run combination in each half of the data. This resulted in 15 separate beta weight estimates for each FIR function in each half of the runs for each surface vertex. The beta weights of all the vertices in a given ROI formed our fMRI response pattern for that ROI. For each ROI and across both halves of the runs, we thus had a total of 30 patterns for each TR and each condition. Note that within a given half of the runs, the 15 patterns were not completely independent of each other, as some of them were estimated from shared runs; however, the patterns between the two halves of the data came from independent runs and were thus completely independent of each other.

To generate a response amplitude time course from each ROI for each condition, we averaged all the beta weights across all the surface vertices within an ROI and from all 30 patterns. The results are shown in Supplementary Figure S2B. Based on the peak responses from all the ROIs and conditions, and to separate encoding and delay signals while accounting for fMRI response lag, we defined an encoding period corresponding to the entire rising part of the response peak (from 4.8 to 8.0 s) and a delay period (from 10.4 to 13.6 s) before the onset of the probe (at 13.6 s). We then averaged the five beta weights within each period to generate an average response for each of these two VWM processing stages.

Prior to our decoding analysis, to remove amplitude differences across categories, ROIs, and VWM processing stages, following our previous studies (Bettencourt & Xu, 2016a; Vaziri-Pashkam & Xu, 2017, 2019; Vaziri-Pashkam et al., 2019; Xu, 2023), we z-normalized each fMRI response pattern. For a given ROI and for a pair of conditions, we used SVM for pattern decoding (LibSVM; Chang & Lin, 2011). We trained a decoder using all the response patterns from one-half of the data (15 patterns) to test its performance on the other half of the data (15 patterns). Training and testing were thus done on independent data sets. We performed training and testing in both directions and averaged the results. We used the binary decoding accuracy to characterize decoding performance, as a simulation in a previous study showed that decoding accuracy closely tracks the underlying signal strength for the majority of the decoding range (from around .52 to .95 of decoding accuracy, with chance-level decoding being .5; see Supplementary Fig. S4 of Xu, 2024). However, distance to the hyperplane also deserves consideration as an alternative measure of decoding performance, as it does not suffer from the ceiling effect inherent to decoding accuracy (i.e., decoding accuracy could never exceed 1). To evaluate whether decoding accuracy or distance to hyperplane better tracks the underlying signal strength, following Xu (2024), we ran a detailed simulation (see Supplementary Results and Supplementary Fig. S3). Our results show that the linear part of the response curve starts later with the distance to hyperplane than with the decoding accuracy measure. In other words, at low signal strength, distance to hyperplane has a larger floor effect than decoding accuracy and is not as sensitive in differentiating signals at low signal strength. Distance to hyperplane is only a better measure than decoding accuracy at very high signal strength when decoding exceeds .95. For the typical signal strength that we encounter in fMRI studies, where decoding accuracy is often less than .9, decoding accuracy is thus a more sensitive measure for low signal strength and is an otherwise equally good measure beyond this signal strength as distance to hyperplane. This justifies our use of decoding accuracy to characterize the decoding performance in the present study.

Training and testing were performed separately for each pair of conditions of interest, and the decoding results were averaged across all relevant pairs of conditions to derive the average decoding performance for a given analysis. To directly compare the different ROIs, to maximize the contrast, and to streamline the analysis, two or three ROIs were grouped together to form sectors based on their anatomical location and response similarity, and the decoding performance was averaged within the ROIs in each sector. These sectors included V1/V2, V3/V4, LOT/VOT, Color-PM (including the posterior and middle color areas), V3a/V3b, IPS0/IPS1, and IPS2/IPS3/IPS4. Within each ROI sector, we performed decoding within each ROI and then averaged the results, rather than forming a combined ROI containing the individual ROIs and then performing decoding. This was done to allow equal weighing of the results from the different ROIs, as size differed across the different ROIs and different participants, decoding based on a combined ROI may bias the results toward the larger ROIs, which could further differ across participants. Our main comparisons focused on the differences among the four ROI sectors. Results from the individual ROIs were reported in Supplementary Figures S4 to S6. We performed two types of decoding.

**Fig. 2.**
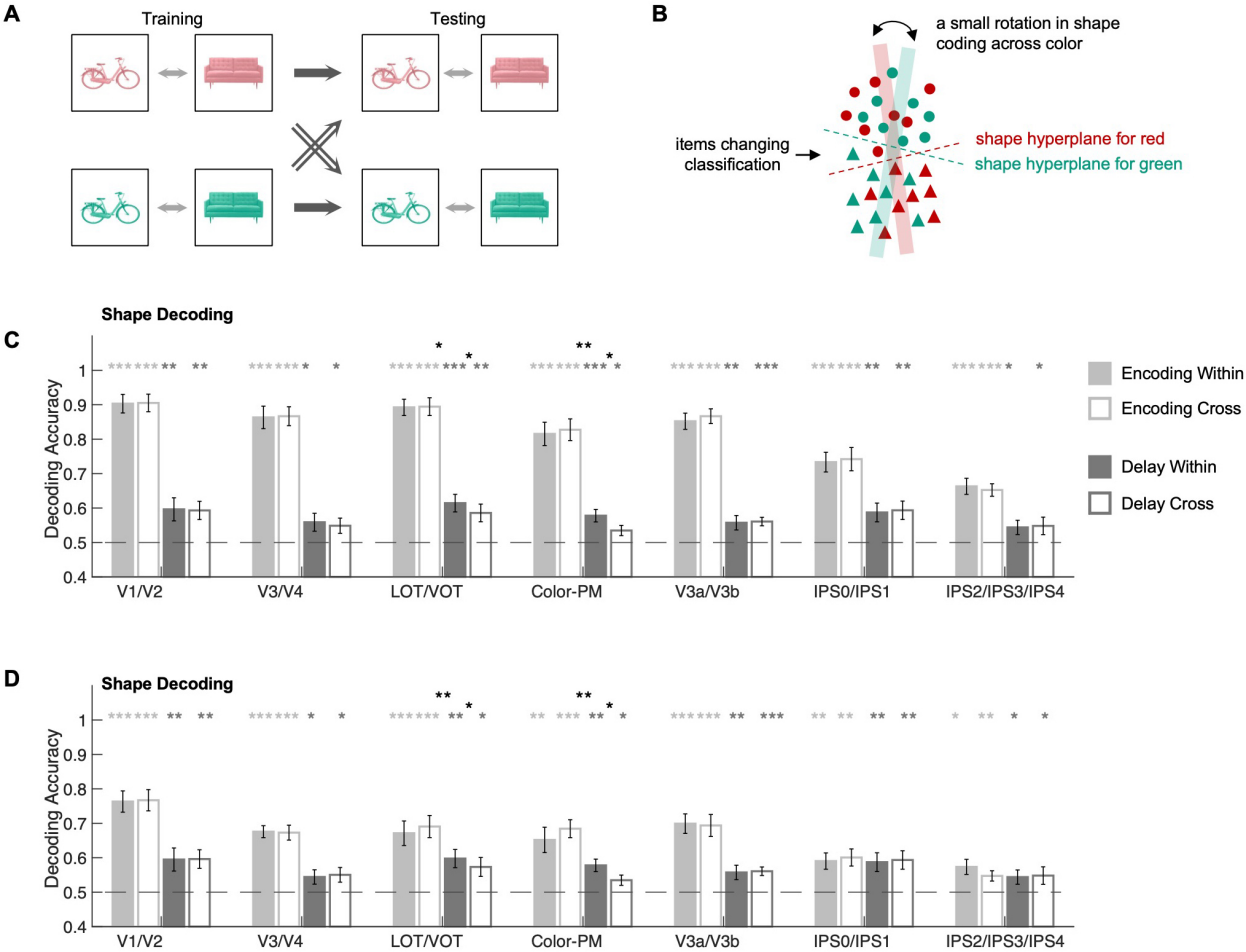
Cross-color shape decoding procedure and results. (A) An illustration of within- and cross-color shape decoding. Here, a linear decoder is trained to discriminate bikes from couches when they were in either red or green; the decoder is then asked to discriminate bikes from couches either in the same color (within-decoding, the solid arrows) or in the other color (cross-decoding, the hollow arrows). (B) A schematic illustration of chance-level color decoding with cross-color shape decoding drop. The two color features are red and green, and the two shape features are dots and triangles. Due to the large overlap in color representation, color decoding is at chance. However, a slight rotation in shape representations across the two colors may still lead to cross-color shape decoding. The transparent elongated bars indicate the axes of the shape representation in each color. (C) Results from the seven pairs of ROI sectors from the encoding period and the delay period. (D) Results from the seven pairs of ROI sectors from the early encoding period and the delay period, with the overlapping color area vertices removed from all the other ROIs. The light grey and medium grey symbols right above the bars mark the significance of each bar compared to chance (.5); the lower row of black symbols mark the significance of the cross-decoding drop in each VWM stage; and the upper row of black symbols mark the significance of the difference in cross-decoding drop between encoding and delay. See Section 2 for more details. Error bars indicate s.e. *p < .05, **p < .01, ***p < .001.

(1) Shape and color within-decoding. For shape decoding, we held the color constant and obtained decoding results for the two objects in each color (i.e., red bike vs. red couch, and green bike vs. green couch). We then averaged the results from the two colors to obtain the average shape decoding performance. For color decoding, we likewise held the object constant and obtained decoding results for the two colors in each object (i.e., red bike vs. green bike, and red couch vs. green couch). We then averaged the results from the two objects to obtain the average color decoding performance. This analysis was done for both the VWM encoding and delay periods. The results are reported in Figure 1D and 1E and Supplementary Figure S4A and S4B.

(2) Cross-color shape decoding. Here, we trained a classifier to decode the two objects in one color and tested its ability to decode the two objects in the other color. We then compared this cross-decoding to within-decoding, in which training and testing were done within the same color (Fig. 2A). We performed this decoding in both directions and averaged the results (i.e., trained the objects in red to decode them in green and vice versa). This analysis was done for both the VWM encoding and delay periods. The results are shown in Figure 2C and Supplementary Figure S6A. We also performed this analysis for each TR, and the results are shown in Supplementary Figure S5.

Color decoding is challenging using colored real-world objects. With a 3T scanner at a resolution of 2 x 2 x 2 mm voxel and using simple shape stimuli matched in saturation and luminance, previous studies have reported robust color decoding throughout OTC during visual perception (e.g., Brouwer & Heeger, 2009; Seymour et al., 2009, 2010; Taylor & Xu, 2022), consistent with findings from neurophysiological studies (see Rentzeperis et al., 2014; Taylor & Xu, 2022). Notably, these fMRI decoding studies all employed a powerful blocked and continuous stimulus presentation paradigm in which the color stimuli filled most of the display (such as concentric rings in Brouwer & Heeger, 2009, logarithmic spirals in Seymour et al., 2010 and Taylor & Xu, 2022, and tessellations in Taylor & Xu, 2022). Even for the handful of fMRI studies that reported color decoding during the VWM delay period at roughly the same fMRI voxel resolution, oriented Gabor gratings were used (Serences et al., 2009; Yu & Shim, 2016). Using colored stimuli that fill most of the display, however, is not feasible for real-world object stimuli. Thus, even with a 3T scanner and at the same 2 x 2 x 2 mm voxel resolution, we expect color decoding performance to reduce substantially for colored real-world objects.

Indeed, in several pilot studies employing a variety of stimulus manipulations, including using real-world objects or their silhouettes appearing in colors matched in saturation and luminance, we found that color decoding for real-world objects was only reliably above chance in the color areas during perception but not for the other OTC areas and not during VWM delay. This poses a significant challenge in using fMRI pattern decoding to document the joint coding of object shape and color in VWM. For example, if we obtain chance-level color decoding and no cross-color shape decoding drop in a brain region, this could be due to the inability of fMRI to detect the presence of color signal and its influence on shape representation, rather than an independent coding of shape and color. We will be unable to draw firm conclusions in this case. However, if we obtain a significant cross-color shape decoding drop despite chance-level color decoding, it would indicate that even though color signals are too weak to differentiate fMRI response patterns above noise, their interaction with object shape signals can, nevertheless, be detected due to object shape decoding being robust (see Fig. 2B for an illustration of such a scenario). Thus, despite the challenge imposed by color decoding from real-world objects, this particular pattern of results would still be informative and allow us to draw definitive conclusions. We will use the presence of this pattern of results to examine interactive shape and color coding in the human brain.

It may be argued that the overall high object shape decoding during VWM encoding could have masked a drop in cross-color shape decoding, even if it existed. To examine this possibility, instead of obtaining the averaged response pattern during the period corresponding to the entire rising part of the response peak spanning five TRs (from 4.8 to 8.0 s), we only took the response pattern from the second TR in this period (at 5.6 s). Additionally, given that both V4 and VOT showed substantial overlap with the color areas, to examine the unique contribution of V4 and VOT, we also excluded the overlapping color areas from both V4 and VOT. The results from the early encoding period and from V4 and VOT without the color area overlap are shown in Figure 2D and Supplementary Figure S6B. While object shape decoding performance was significantly reduced during the VWM encoding period, our main effects remained consistent, and the statistical evidence was, if anything, stronger.

Given that the two types of objects used in the present study did not share the exact shape spatial envelope (unlike the stimuli used in Taylor & Xu, 2022), a cross-shape color decoding would be confounded with spatial envelope differences between the two types of objects. That is, a failure to decode color across different shapes could be entirely due to different/non-overlapping neuronal populations being activated for the two objects due to their spatial coverage differences, rather than the integration of shape and color. Thus, to examine integrated shape and color representations, only cross-color shape decoding was considered here. Furthermore, as the results will show, color decoding was very weak in the present study, adding another reason not to examine cross-shape color decoding.

(3) Pattern-difference decoding. Instead of using a cross-decoding approach, following Taylor and Xu (2022, 2024), we also directly examined changes in patterns as a result of interactive shape and color coding. Specifically, we first obtain a difference pattern between the patterns of red bikes and red couches (d1) and a difference pattern between the patterns of green bikes and green couches (d2). We then tried to decode d1 and d2 following the decoding procedure described above. An above-chance level of decoding would indicate that the difference patterns differed between these two pairs of conditions, indicating interactive coding. We have found, in general, consistencies between the cross-decoding and pattern-difference decoding results (Taylor & Xu, 2022, 2024). This approach is more sensitive to detect interactive coding when the within-decoding performance is near the ceiling, as a change in response pattern may not lead to a cross-decoding drop as long as the pattern still stays on the correct side of the decision hyperplane, whereas such a change could lead to significant pattern-difference decoding. This approach could thus potentially be more effective in detecting interactive coding during the VWM encoding period when the object shape within-decoding performance is high. The results of this analysis are shown in Supplementary Figure S7.

#### 2.5.3 Statistical analyses

T-tests were used to assess within and cross-decoding performance against chance (one sample, 1-tailed, as only effects above chance were meaningful), and cross-decoding drop (paired, 1-tailed, as the effect was expected to be either null or with cross-decoding being lower than within-decoding). Correction for multiple comparisons was applied using the Benjamini-Hochberg method (Benjamini & Hochberg, 1995) for the number of tests of the same type performed within each ROI or sector. In Figure 1D and 1E and Supplementary Figures S4A, S4B, and S7, corrections were applied to two tests performed in each ROI or ROI sector. In Figure 2C and 2D, and Supplementary Figure S6A and S6B, for decoding performance against chance, corrections were applied to four tests performed in each ROI or ROI sector, and for cross-decoding drop against chance, corrections were applied to two tests performed in each ROI or ROI sector. Repeated measures ANOVAs were used to assess main effects and interactions between variables of interest.

## 3 Results

In this study, human participants maintained colored objects in VWM (Fig. 1A). We used two types of objects, bikes and couches, which appeared in two colors, red and green. To increase task difficulty and ensure that a visual code was employed to retain VWM representation, we used similar-looking exemplars of a given object in different shades of a given color. Each trial contained two different targets, both exemplars of the same object in the same shade of a color. For a “same” response, the probe object at the end of the VWM delay was one of the target exemplars in the same shade of color; and for a “different” response, the probe object was either one of the target exemplars in a different shade of the same color or a new exemplar of the same object in the same shade of color (Fig. 1A and 1B). Responses were examined across OTC and PPC (Fig. 1C). From the averaged fMRI response time courses of each ROI, the encoding and delay periods were defined, fMRI responses were averaged within each period, and fMRI response patterns were generated for each ROI and each condition (see Section 2). To increase power and streamline the analysis, we grouped ROIs together to form sectors based on anatomical location and response similarity, with the decoding performance averaged within the ROIs in each sector. These sectors included V1/V2, V3/V4, LOT/VOT, Color-PM (including the posterior and middle color areas), V3a/V3b, IPS0/IPS1, and IPS2/IPS3/IPS4.

The mean (and standard deviation) of behavioral performance accuracy, averaged over shape change, color change, and no change trials, was .80 (.11). For the shape change trials, accuracies for the bike and couch trials were .82 (.18) and .85 (.19), respectively, with no difference between the two, *t(11)* = 1.28 *p* = .22. For the color change trials, accuracies for the red and green trials were .81 (.12) and .58 (.21), respectively, with performance being higher for the red than green trials, *t(11)* = 6.49, *p* < .001. Although each set of six hues for a given color (red or green) was evenly spaced, with the red and green hues on opposite sides of the color wheel (i.e., 180° apart) in the CIELUV color space, it appeared to be more difficult to retain distinctive representations for the different hues of green than those of red in VWM. In the present study, while the VWM task was performed at the exemplar level (i.e., retaining the precise object exemplar and the exact hue of color), decoding was performed at the category level by pooling together the visual code for the different exemplars of the same object or different hues of the same color. This followed the design of Harrison and Tong (2009) to ensure that a visual code, rather than a verbal code, was retained in VWM. Thus, whether or not the different hues of a color would form equally distinctive representations in VWM should not impact red vs. green color decoding.

### 3.1 Shape and color decoding

Significant shape decoding was found during both VWM encoding and delay periods, with decoding performance being greater for the encoding than the delay period (Fig. 1D; see the asterisks marking the significance levels; 1-tailed and corrected, see Section 2 for more details; see Supplementary Figure S4A for the results of the individual ROIs). These results are consistent with prior reports showing significant representations of object shape information during both the VWM encoding and delay periods throughout OTC and PPC areas.

Unlike shape coding, significant color decoding was found only in LOT/VOT and Color-PM sectors during VWM encoding but in none of the ROI sectors during VWM delay (Fig. 1E; 1-tailed and corrected; see the asterisks marking the significance levels). A few individual ROIs did show significant or marginally significant color decoding during VWM delay, in addition to showing significant color decoding during VWM encoding (Supplementary Fig. S4B). These results show that color information was coded during perception in higher visual regions regardless of their univariate response preference to shape or color, consistent with the results of Taylor and Xu (2022). Meanwhile, color decoding was overall weak, especially during the VWM delay.

### 3.2 Cross-color object shape coding

Prior neurophysiology and fMRI research have reported the existence of color representation throughout OTC during visual perception in the primate brain. Even though color signals were too weak to robustly differentiate fMRI response patterns in the present study above the noise level, if a significant shape and color interaction exists, such an interaction might still modulate the robust object shape decoding as illustrated in Figure 2B.

To test this, as illustrated in Figure 2A, we trained a linear classifier to decode two object shapes in one color and then asked the classifier to decode the same two object shapes either in the same color (within-decoding) or in another color (cross-decoding). The results are shown in Figure 2C (see the asterisks marking the significance levels; 1-tailed and corrected, see Section 2 for more details; see Supplementary Fig. S5 for decoding for each TR and Supplementary Figure S6A for decoding of each individual ROI). We found significantly above-chance cross-decoding in all the ROI sectors and almost all the individual ROIs. We then compared the drop in cross-decoding to within-decoding. During VWM encoding, even though above-chance color decoding was present in both LOT/VOT and Color-PM, there was no cross-color shape decoding drop. Interestingly, during the VWM delay period, despite the absence of color decoding in any of the ROI sectors, we obtained a significant cross-color shape decoding drop in LOT/VOT and Color-PM. Moreover, repeated measure ANOVA in both LOT/VOT and Color-PM showed a main effect of VWM processing stage (encoding vs. delay) (*Fs* > 88.37, *ps* < .001), no effect of cross-decoding (*Fs* < 2.36, *ps* > .15), but a significant interaction between VWM stage and cross-decoding (*Fs* > 7.42, *ps* < .020). Further pairwise comparisons showed that the amount of cross-decoding drop was greater during VWM delay than encoding for both LOT/VOT and Color-PM (*ts* > 2.72, *ps* < .020; two-tailed).

It may be argued that the overall high object shape decoding during VWM encoding could have masked a drop in cross-color shape decoding, even if it existed. To examine this possibility, in a new analysis, instead of obtaining the averaged response pattern during the encoding period corresponding to the entire rising part of the response peak spanning several TRs (see Supplementary Fig. S2B), we only took the response pattern from the second TR in this period. Because VOT overlapped substantially with the color areas (31% on average; see Fig. 1C), one may argue that the significant cross-color shape decoding drop observed during VWM delay in LOT/VOT could have entirely come from the overlapping color area vertices. To test this, in the new analysis, we also redefined each ROI by excluding all overlapping color area vertices.

As shown in Figure 2D (see the asterisks marking the significance levels; 1-tailed and corrected; see Supplementary Fig. S6B for the results of the individual ROIs), using responses from the early delay period resulted in a significant reduction in object shape decoding during the VWM encoding period. Nevertheless, even with the color area vertices removed from LOT/VOT, we still observed significantly above-chance cross-color shape decoding in all the ROI sectors and in almost all the individual ROIs. Importantly, we again saw a significant drop in cross-color shape decoding compared to within-decoding during the VWM delay, but not during encoding, in both LOT/VOT and Color-PM. We also noted a significant, if not stronger, interaction between the VWM processing stage and within vs. cross-decoding (main effect of VWM stage, *Fs* > 5.51, *ps* < .039; main effect of cross-decoding, *Fs* < .12, *ps* > .74; and the interaction between the two, *Fs* > 10.41, *ps* < .008). Further pairwise comparisons showed that the amount of cross-decoding drop was greater during VWM delay than encoding for both LOT/VOT and Color-PM (*ts* > 3.22, *ps* < .008; 2-tailed).

In addition to using a cross-decoding approach, following Taylor and Xu (2022, 2024), we also performed pattern-difference decoding to directly examine changes in patterns as a result of interactive shape and color coding (i.e., decoding the differences in patterns from pairs of conditions, see Section 2). Above-chance decoding would indicate that the difference patterns were not the same between pairs of conditions, indicating interactive coding. This approach is more sensitive to detect interactive coding when the within-decoding performance is near ceiling, as a change in response pattern may not lead to a cross-decoding drop as long as the pattern still stays on the correct side of the decoding decision hyperplane, whereas such a change could lead to significant pattern-difference decoding (see Taylor & Xu, 2024 for a detailed illustration). This approach could thus be more sensitive in detecting interactive coding during the VWM encoding period when the object shape within-decoding performance is high. Even with this approach, however, we found no significant pattern-difference decoding during VWM encoding (Supplementary Fig. S7A). Despite this approach operating on the differences between patterns, which could result in effects being masked by noise when the differences are small, we still obtained some marginally significant pattern-difference decoding during VWM delay in Color-PM, consistent with our cross-decoding results (Supplementary Fig. S7B).

## 4 Discussion

Using colored real-world objects and fMRI pattern cross-decoding analyses, we found that, during the VWM perceptual encoding period, object shape and color representations were independent, such that a linear classifier trained to decode two object shapes in one color can generalize its performance to the same two objects in another color with no drop in performance. This is consistent with our prior findings from perceptual tasks (Taylor & Xu, 2022). However, during the VWM delay period, cross-decoding—although still significant—dropped in object shape-selective (LOT/VOT) and color-selective areas (Color-PM). This indicates that object shape and color representations were partially integrated and interacted with each other in these two brain regions during VWM delay. To our knowledge, this is the first time that interactive shape and color representations have been reported in VWM using fMRI pattern decoding in the human brain. Given the evidence that VWM signals from OTC are largely driven by feedback from areas such as PFC and PPC (e.g., Lawrence et al., 2018; Liebe et al., 2012; van Kerkoerle et al., 2017; Xu, 2023), a change from independent to partially interactive object shape and color representations between VWM encoding and delay is likely driven by feedback signals during the VWM consolidation period. This also shows that VWM representations in sensory areas are not mere extensions of their perceptual representations, as proposed by the sensory account of VWM (e.g., Christophel et al., 2017; D’Esposito and Postle, 2015; Serences, 2016). Rather, the present study joins a host of recent findings reporting that significant representational transformations can occur in VWM (Duan & Curtis, 2024; Kwak & Curtis, 2022; Li & Curtis, 2023; Xu, 2023, 2025; Yan et al., 2023; see Kiyonaga & Serences, in press, for an extended review) and further elucidates the limitations of the sensory account in providing a full understanding of VWM representations (see also Leavitt et al., 2017; Xu, 2017, 2018, 2020, 2021).

We recently showed that different streams of information could be maintained independently in VWM to reduce interference from concurrently active visual representations, whether such interference comes from different targets stored together in VWM or from the presence of distractors during the delay period (Xu, 2024). Thus, it is not the case that VWM cannot maintain independent feature representations. On the contrary, given that transformed VWM representations in sensory areas have been shown to better meet the demands of VWM tasks (e.g., tracking task-relevant representations in PPC, Xu, 2023; forming more streamlined representations, Kwak & Curtis, 2022; mediating behavior, Li & Curtis, 2023), a partially integrated object shape and color representation could likewise facilitate task-relevant information storage in VWM. That is, with an object’s color and shape being features of the same object, rather than sustaining two independent representations, VWM could partially integrate shape and color representations to help retain both features at the same time. This could be accomplished by rotating representation in the shape and color representational space, as depicted in Figure 2B. Such a rotation could make each conjunction more distinctive, yet at the same time represent in close proximity the shared shape in the different conjunctions, enabling successful cross-color shape decoding. Such a representational scheme may mediate the well-known object-based storage benefit found in behavioral VWM studies (e.g., Luck & Vogel, 1997; Xu, 2002a, 2002b). Future research is needed to test such a representational scheme and document how the magnitude of the cross-decoding drop may be directly linked to the change in the feature representational space in VWM. In the present study, while the VWM task was performed at the exemplar level (i.e., retaining the precise object exemplar and the exact shade of color), decoding was performed at the category level by pooling together the visual code for the different exemplars of the same object or different hues of the same color. This was done following the design of Harrison and Tong (2009) and was necessary to ensure that a visual code, rather than a verbal code, was retained in VWM. Nonetheless, our task resulted in a heightened VWM storage demand, and this raised an interesting question regarding how storage demand may affect the presence of interactive shape and color coding. One option is that interactive coding occurs in the OTC during the WM delay regardless of storage demand. Another option is that an increase in storage demand may prompt top-down control and feedback mechanisms to integrate shape and color representations in OTC to ensure better retention of their conjunctions. It would, thus, be interesting in future research to systematically vary the VWM storage load and chart the detailed relationship between interactive coding and storage load.

Given that the ultimate goal of vision research is to understand information storage in VWM in everyday vision, we used real-world complex objects in the present study. The usage of such objects also increased our chance of detecting any representational change, given that an orthogonal shape and color coding in perception is more prominent for complex than simple shapes (Taylor & Xu, 2022). However, as real-world stimuli do not typically fill the entire display areas, unlike the simple artificial stimuli used in previous studies (e.g., perception studies - Brouwer & Heeger, 2009; Seymour et al., 2009, 2010; Taylor & Xu, 2022; and VWM studies - Serences et al., 2009; Yu & Shim, 2017), the usage of real-world objects here likely contributed to the low or chance-level color decoding performance during the VWM encoding and delay periods. This complicates the interpretation of results, as a lack of cross-color shape decoding drop could simply be due to the inability of fMRI to detect the presence of color signal and its influence on shape representation, rather than reflecting how object shape and color are coded together. However, even though color signals might be too weak to robustly differentiate fMRI response patterns above noise, their interactions with object shape signals could, nevertheless, modulate object shape decoding, given the latter’s strong signal strength, as illustrated in Figure 2B (see also Section 2). This could explain why we obtained a significant cross-color shape decoding drop in higher object shape and color areas in OTC, despite a lack of significant color decoding during VWM delay.

Meanwhile, it should be noted that the effect of interactive coding as measured by cross-decoding is relatively small. Moreover, it remains unknown in other brain regions how an object’s shape and color may be coded together in VWM. The success of fMRI pattern decoding relies on the existence of heterogeneous neuronal populations selective for different features distributed at a resolution visible by fMRI. It is very possible that the color-selective neurons are distributed more homogenously at our fMRI voxel resolution, given our MRI field strength, resulting in weak but still significant interactive effects in some regions and the absence of any effects in the other regions. A smaller voxel size and/or a higher field strength in future fMRI studies or a neurophysiological approach are, thus, needed to replicate and extend the present results. Given that an object’s shape and color are two of the most defining features of a real-world object, despite the challenges, understanding the neural underpinnings of their representations in the human brain is a worthy and rewarding endeavor for future research.

## Supplementary Material

Supplementary Material

## Data Availability

Data reported in this study are available at https://osf.io/j6kcm/.

## References

[IMAG.a.1049-b1] Benjamini, Y., & Hochberg, Y. (1995). Controlling the false discovery rate: A practical and powerful approach to multiple testing. Journal of the Royal Statistical Society: Series B (Methodological), 57(1), 289–300. 10.1111/j.2517-6161.1995.tb02031.x

[IMAG.a.1049-b2] Bettencourt, K., & Xu, Y. (2013). The role of transverse occipital sulcus in scene perception and its relationship to object individuation in inferior intraparietal sulcus. Journal of Cognitive Neuroscience, 25(10), 1711–1722. 10.1162/jocn_a_00422 23662863 PMC3758388

[IMAG.a.1049-b3] Bettencourt, K. C., & Xu, Y. (2016a). Decoding under distraction reveals distinct occipital and parietal contributions to visual short-term memory representation. Nature Neuroscience, 19(1), 150–157. 10.1038/nn.4174 26595654 PMC4696876

[IMAG.a.1049-b4] Bettencourt, K. C., & Xu, Y. (2016b). Understanding location- and feature-based processing along the human intraparietal sulcus. Journal of Neurophysiology, 116(3), 1488–1497. 10.1152/jn.00404.2016 27440243 PMC5040374

[IMAG.a.1049-b5] Brainard, D. H. (1997). The psychophysics toolbox. Spatial Vision, 10(4), 433–436. 10.1163/156856897x00357 9176952

[IMAG.a.1049-b6] Brodoehl, S., Gaser, C., Dahnke, R., Witte, O. W., & Klingner, C. M. (2020). Surface-based analysis increases the specificity of cortical activation patterns and connectivity results. Scientific Reports, 10(1), 5737. 10.1038/s41598-020-62832-z 32235885 PMC7109138

[IMAG.a.1049-b7] Brouwer, G. J., & Heeger, D. J. (2009). Decoding and reconstructing color from responses in human visual cortex. Journal of Neuroscience, 29(44), 13992–14003. 10.1523/jneurosci.3577-09.2009 19890009 PMC2799419

[IMAG.a.1049-b8] Chang, C. C., & Lin, C. J. (2011). LIBSVM: A library for support vector machines. ACM Transactions on Intelligent Systems and Technology, 2(3), 1–27. 10.1145/1961189.1961199

[IMAG.a.1049-b9] Christophel, T. B., Klink, P. C., Spitzer, B., Roelfsema, P. R., & Haynes, J. D. (2017). The distributed nature of working memory. Trends in Cognitive Sciences, 21(2), 111–124. 10.1016/j.tics.2016.12.007 28063661

[IMAG.a.1049-b10] Dale, A. M., Fischl, B., & Sereno, M. I. (1999). Cortical surface-based analysis: I. Segmentation and surface reconstruction. NeuroImage, 9(2), 179–194. 10.1006/nimg.1998.0395 9931268

[IMAG.a.1049-b11] D’Esposito, M., & Postle, B. R. (2015). The cognitive neuroscience of working memory. Annual Review of Psychology, 66, 115–142. 10.1146/annurev-psych-010814-015031 PMC4374359 25251486

[IMAG.a.1049-b12] Duan, Z., & Curtis, C. E. (2024). Visual working memories are abstractions of percepts. eLife, *13*, RP94191. 10.7554/elife.94191 PMC11147505 38819426

[IMAG.a.1049-b13] Grill-Spector, K., Kushnir, T., Edelman, S., Itzchak, Y., & Malach, R. (1998). Cue-invariant activation in object-related areas of the human occipital lobe. Neuron, 21(1), 191–202. 10.1016/s0896-6273(00)80526-7 9697863

[IMAG.a.1049-b14] Harrison, S. A., & Tong, F. (2009). Decoding reveals the contents of visual working memory in early visual areas. Nature, 458(7238), 632–635. 10.1038/nature07832 19225460 PMC2709809

[IMAG.a.1049-b15] Jeong, S. K., & Xu, Y. (2017). Task-context dependent linear representation of multiple visual objects in human parietal cortex. Journal of Cognitive Neuroscience, 29(11), 1778–1789. 10.1162/jocn_a_01156 28598733

[IMAG.a.1049-b16] Kiyonaga, A., & Serences, J. T. Sensory reformatting for a working visual memory. Trends in Cognitive Sciences (In press). 10.1016/j.tics.2025.09.006 PMC12782179 41067992

[IMAG.a.1049-b17] Kourtzi, Z., & Kanwisher, N. (2000). Cortical regions involved in perceiving object shape. Journal of Neuroscience, 20(9), 3310–3318. 10.1523/jneurosci.20-09-03310.2000 10777794 PMC6773111

[IMAG.a.1049-b18] Kwak, Y., & Curtis, C. E. (2022). Unveiling the abstract format of mnemonic representations. Neuron, 110(11), 1822–1828. 10.1016/j.neuron.2022.03.016 35395195 PMC9167733

[IMAG.a.1049-b19] Lafer-Sousa, R., Conway, B. R., & Kanwisher, N. G. (2016). Color-biased regions of the ventral visual pathway lie between face- and place-selective regions in humans, as in macaques. Journal of Neuroscience, 36(5), 1682–1697. 10.1523/jneurosci.3164-15.2016 26843649 PMC4737777

[IMAG.a.1049-b20] Lawrence, S. J. D., van Mourik, T., Kok, P., Koopmans, P. J., Norris, D. G., & de Lange, F. P. (2018). Laminar organization of working memory signals in human visual cortex. Current Biology, 28(21), 3435–3440. 10.1016/j.cub.2018.08.043 30344121

[IMAG.a.1049-b21] Leavitt, M. L., Mendoza-Halliday, D., & Martinez-Trujillo, J. C. (2017). Sustained activity encoding working memories: Not fully distributed. Trends in Neurosciences, 40(6), 328–346. 10.1016/j.tins.2017.04.004 28515011

[IMAG.a.1049-b22] Li, H. H., & Curtis, C. E. (2023). Neural population dynamics of human working memory. Current Biology, 33(18), 3775–3784. 10.1016/j.cub.2023.07.067 37595590 PMC10528783

[IMAG.a.1049-b23] Liebe, S., Hoerzer, G. M., Logothetis, N. K., & Rainer, G. (2012). Theta coupling between V4 and prefrontal cortex predicts visual short-term memory performance. Nature Neuroscience, 15(3), 456–464. 10.1038/nn.3038 22286175

[IMAG.a.1049-b24] Luck, S. J., & Vogel, E. K. (1997). The capacity of visual working memory for features and conjunctions. Nature, 390(6657), 279–281. 10.1038/36846 9384378

[IMAG.a.1049-b25] Malach, R., Reppas, J. B., Benson, R. R., Kwong, K. K., Jiang, H., Kennedy, W. A., Ledden, P. J., Brady, T. J., Rosen, B. R., & Tootell, R. B. (1995). Object-related activity revealed by functional magnetic resonance imaging in human occipital cortex. Proceedings of the National Academy of Sciences, 92(18), 8135–8139. 10.1073/pnas.92.18.8135 PMC41110 7667258

[IMAG.a.1049-b26] Mocz, V., Jeong, S. K., Chun, M., & Xu, Y. (2023). The representation of multiple visual objects in human ventral visual areas and in convolutional neural networks. Scientific Reports, 13(1), 9088. 10.1038/s41598-023-36029-z 37277406 PMC10241785

[IMAG.a.1049-b27] Oosterhof, N. N., Wiestler, T., Downing, P. E., & Diedrichsen, J. (2011). A comparison of volume-based and surface-based multi-voxel pattern analysis. NeuroImage, 56(2), 593–600. 10.1016/j.neuroimage.2010.04.270 20621701

[IMAG.a.1049-b28] Orban, G. A., Van Essen, D., & Vanduffel, W. (2004). Comparative mapping of higher visual areas in monkeys and humans. Trends in Cognitive Sciences, 8(7), 315–324. 10.1016/j.tics.2004.05.009 15242691

[IMAG.a.1049-b29] Rentzeperis, I., Nikolaev, A. R., Kiper, D. C., & van Leeuwen, C. (2014). Distributed processing of color and form in the visual cortex. Frontiers in Psychology, 5, 932. 10.3389/fpsyg.2014.00932 25386146 PMC4209824

[IMAG.a.1049-b200] Serences, J. T. (2016). Neural mechanisms of information storage in visual short-term memory. Vision Research, 128, 53–67. 10.1016/j.visres.2016.09.010 27668990 PMC5079778

[IMAG.a.1049-b30] Serences, J. T., Ester, E. F., Vogel, E. K., & Awh, E. (2009). Stimulus-specific delay activity in human primary visual cortex. Psychological Science, 20(2), 207–214. 10.1111/j.1467-9280.2009.02276.x 19170936 PMC2875116

[IMAG.a.1049-b31] Sereno, M. I., Dale, A. M., Reppas, J. B., Kwong, K. K., Belliveau, J. W., Brady, T. J., Rosen, B. R., & Tootell, R. B. (1995). Borders of multiple visual areas in humans revealed by functional magnetic resonance imaging. Science, 268(5212), 889–893. 10.1126/science.7754376 7754376

[IMAG.a.1049-b32] Seymour, K., Clifford, C. W., Logothetis, N. K., & Bartels, A. (2009). The coding of color, motion, and their conjunction in the human visual cortex. Current Biology, 19(3), 177–183. 10.1016/j.cub.2008.12.050 19185496

[IMAG.a.1049-b33] Seymour, K., Clifford, C. W., Logothetis, N. K., & Bartels, A. (2010). Coding and binding of color and form in visual cortex. Cerebral Cortex, 20(8), 1946–1954. 10.1093/cercor/bhp265 20019147

[IMAG.a.1049-b34] Swisher, J. D., Halko, M. A., Merabet, L. B., McMains, S. A., & Somers, D. C. (2007). Visual topography of human intraparietal sulcus. Journal of Neuroscience, 27(20), 5326–5337. 10.1523/jneurosci.0991-07.2007 17507555 PMC6672354

[IMAG.a.1049-b35] Taylor, J., & Xu, Y. (2021). Joint representation of color and shape in convolutional neural networks: A stimulus-rich network perspective. PLoS One, 16(7), e0253442. 10.1371/journal.pone.0253442 34191815 PMC8244861

[IMAG.a.1049-b36] Taylor, J., & Xu, Y. (2022). Representation of color, form, and their conjunction across the human ventral visual pathway. NeuroImage, 251, 118941. 10.1016/j.neuroimage.2022.118941 35122966 PMC9014861

[IMAG.a.1049-b37] Taylor, J., & Xu, Y. (2024). Using fMRI to examine nonlinear mixed selectivity tuning to task and category in the human brain. Imaging Neuroscience, 2, 1–21. 10.1162/imag_a_00354 PMC12176419 40534624

[IMAG.a.1049-b38] van Kerkoerle, T., Self, M. W., & Roelfsema, P. R. (2017). Layer-specificity in the effects of attention and working memory on activity in primary visual cortex. Nature Communications, 8, 13804. 10.1038/ncomms13804 PMC5227065 28054544

[IMAG.a.1049-b39] Vaziri-Pashkam, M., Taylor, J., & Xu, Y. (2019). Spatial frequency tolerant visual object representations in the human ventral and dorsal visual processing pathways. Journal of Cognitive Neuroscience, 31(1), 49–63. 10.1162/jocn_a_01335 30188780

[IMAG.a.1049-b40] Vaziri-Pashkam, M., & Xu, Y. (2017). Goal-directed visual processing differentially impacts human ventral and dorsal visual representations. Journal of Neuroscience, 37(36), 8767–8782. 10.1523/jneurosci.3392-16.2017 28821655 PMC5588467

[IMAG.a.1049-b41] Vaziri-Pashkam, M., & Xu, Y. (2019). An information-driven 2-pathway characterization of occipitotemporal and posterior parietal visual object representations. Cerebral Cortex, 29(5), 2034–2050. 10.1093/cercor/bhy080 29659730 PMC7302692

[IMAG.a.1049-b42] Xu, Y. (2002a). Limitations of object-based feature encoding in visual short-term memory. Journal of Experimental Psychology: Human Perception and Performance, 28(2), 458–468. 10.1037//0096-1523.28.2.458 11999866

[IMAG.a.1049-b43] Xu, Y. (2002b). Encoding color and shape from different parts of an object in visual short-term memory. Perception & Psychophysics, 64(7), 1260–1280. 10.3758/bf03194770 12519024

[IMAG.a.1049-b44] Xu, Y. (2017). Reevaluating the sensory account of visual working memory storage. Trends in Cognitive Sciences, 21(10), 794–815. 10.1016/j.tics.2017.06.013 28774684

[IMAG.a.1049-b45] Xu, Y. (2018). Sensory cortex is nonessential in working memory storage: A reply to commentaries. Trends in Cognitive Sciences, 22(3), 192–193. 10.1016/j.tics.2017.12.008 29482822

[IMAG.a.1049-b46] Xu, Y. (2020). Revisit once more the sensory storage account of visual working memory. Visual Cognition, 28(5–8), 433–436. 10.1080/13506285.2020.1818659 33841024 PMC8034609

[IMAG.a.1049-b47] Xu, Y. (2021). Towards a better understanding of information storage in visual working memory. Visual Cognition, 29(7), 437–445. 10.1080/13506285.2021.1946230 35496937 PMC9053365

[IMAG.a.1049-b48] Xu, Y. (2023). Parietal-driven visual working memory representation in occipito-temporal cortex. Current Biology, 33(21), 4516–4523. 10.1016/j.cub.2023.08.080 37741281 PMC10615870

[IMAG.a.1049-b49] Xu, Y. (2024). The human posterior parietal cortices orthogonalize the representation of different streams of information concurrently coded in visual working memory. PLoS Biology, 22(3), e3002915. 10.1371/journal.pbio.3002915 39570984 PMC11620661

[IMAG.a.1049-b50] Xu, Y. (2025). Transformed visual working memory representations in human occipitotemporal and posterior parietal cortices. Eneuro, 12(7), ENEURO.0162-25.2025. 10.1523/eneuro.0162-25.2025 40473470 PMC12243946

[IMAG.a.1049-b51] Xu, Y., & Jeong, S. K. (2015). The contribution of human superior intraparietal sulcus to visual short-term memory and perception. In P. Jolicoeur, C. Lefebvre, & J. Martinez-Trujillo (Eds.), Mechanisms of sensory working memory: Attention and performance XXV (Vol. 1, pp. 33–42). Academic Press. 10.1016/b978-0-12-801371-7.00004-1

[IMAG.a.1049-b52] Xu, Y., & Vaziri-Pashkam, M. (2019). Task modulation of the 2-pathway characterization of occipitotemporal and posterior parietal visual object representations. Neuropsychologia, 132, 107140. 10.1016/j.neuropsychologia.2019.107140 31301350 PMC6857731

[IMAG.a.1049-b53] Xu, Y., & Vaziri-Pashkam, M. (2022). Understanding transformation tolerant visual object representations in the human brain and convolutional neural networks. NeuroImage, 263, 119635. 10.1016/j.neuroimage.2022.119635 36116617 PMC11283825

[IMAG.a.1049-b54] Yan, C., Christophel, T. B., Allefeld, C., & Haynes, J. (2023). Categorical working memory codes in human visual cortex. NeuroImage, 274, 120149. 10.1016/j.neuroimage.2023.120149 37191658

[IMAG.a.1049-b55] Yu, Q., & Shim, W. M. (2017). Occipital, parietal, and frontal cortices selectively maintain task-relevant features of multi-feature objects in visual working memory. NeuroImage, 157, 97–107. 10.1016/j.neuroimage.2017.05.055 28559190

[IMAG.a.1049-b56] Zhou, B., Lapedriza, A., Khosla, A., Oliva, A., & Torralba, A. (2018). Places: A 10 million image database for scene recognition. IEEE Transactions on Pattern Analysis and Machine Intelligence, 40(6), 1452–1464. 10.1109/tpami.2017.2723009 28692961

