# Supplementary material for: Interactive shape and color representation in visual working memory for colored objects in the human occipitotemporal cortex
Source: Imaging Neurosci (Camb). 2025 Dec 5;3:IMAG.a.1049. doi: 10.1162/IMAG.a.1049 (PMC13288494; doi:10.1162/IMAG.a.1049)
Supplement: Supplementary Material [file IMAG.a.1049_supp.pdf]

Supplementary Results

Simulations on examining decoding accuracy and distance to hyperplane as a function of signal strength

Using a simulation, Xu (2024) showed that once SVM decoding rises above chance (.5), starting at 5% signal strength (with a decoding accuracy of about .51-.52), a linear relationship holds between decoding accuracy and the underlying signal strength, up to very high signal strengths (when decoding exceeds .95). One could use a probit transformation (i.e., transformation by the inverse of the normal function) to further straighten the decoding accuracies to make them more linear, but very similar results were obtained for the raw decoding accuracies and for the probit-transformed decoding accuracies (see Xu, 2024). Besides decoding accuracy, one could also measure the distance to the SVM decision hyperplane as another way to characterize decoding performance. Is distance to hyperplane a better measure than decoding accuracy?

To address this question, we followed the simulation procedure in Xu (2024) and examined how decoding accuracy and distance to hyperplane track the underlying signal strength. Specifically, two random patterns, A and B, were first created, each containing 1500 units (chosen to match the lower-end number of vertices in the brain ROIs, see Supplementary Figure 2A). Decoding of A vs B was then performed at different levels of signal strength, from decoding 1% of A and B to decoding 40% of A and B, at a 1% increment (decoding accuracy approached 1 at 20% of signal strength). For each decoding analysis, to generate sufficient patterns, 16 samples for A and 16 samples for B were created to mimic the data obtained in a typical fMRI decoding study. At a given signal level of *s*, a pattern for A would be generated by adding *s%* of A and *(1-s)%* of a randomly generated noise pattern, with the noise pattern being different for each of the 16 A and B samples. Decoding was then performed on these 16 A and 16 B patterns. For a given pair of patterns A and B at a given signal level, this simulation was repeated 1,000 times, and the results were averaged. We measured both decoding accuracies and distances to the hyperplane. Signed distances were used in the

calculation, such that points on the wrong side of the decision hyperplane were given negative values.

Supplementary Figure 3 shows 10 simulation cases, each starting with two new randomly initialized A and B patterns. As in Xu (2024), aside from a small floor effect at the extremely low signal strength, decoding accuracy linearly tracks the underlying signal strength, starting at around 5% signal strength (with a decoding accuracy of about .51-.52) up until decoding reaches around .95, when a nonlinear relationship returns due to a ceiling effect. Compared to decoding accuracy, distance to hyperplane has a larger floor effect and is not able to linearly track the increase in signal strength until the signal is around 15% or more. The reason distance to hyperplane performs poorly at low signal strength could be that this measure introduces additional variance to the measure (e.g., the measure could never be exactly zero even with chance-level performance), making it harder to detect the signal when it is close to the noise level. Meanwhile, distance to hyperplane is a better measure than decoding accuracy beyond 95% signal strength when SVM decoding reaches the ceiling. Thus, distance to hyperplane is only a better measure than decoding accuracy at very high signal strength. For the typical signal strengths encountered in fMRI studies, where decoding is less than .9, decoding accuracy is thus a more sensitive measure for low signal strength and is an otherwise equally good measure at other signal strengths as distance to hyperplane. Overall, for the range of decoding performance encountered in fMRI studies, decoding accuracy appears to be a better measure than distance to hyperplane.

A

B

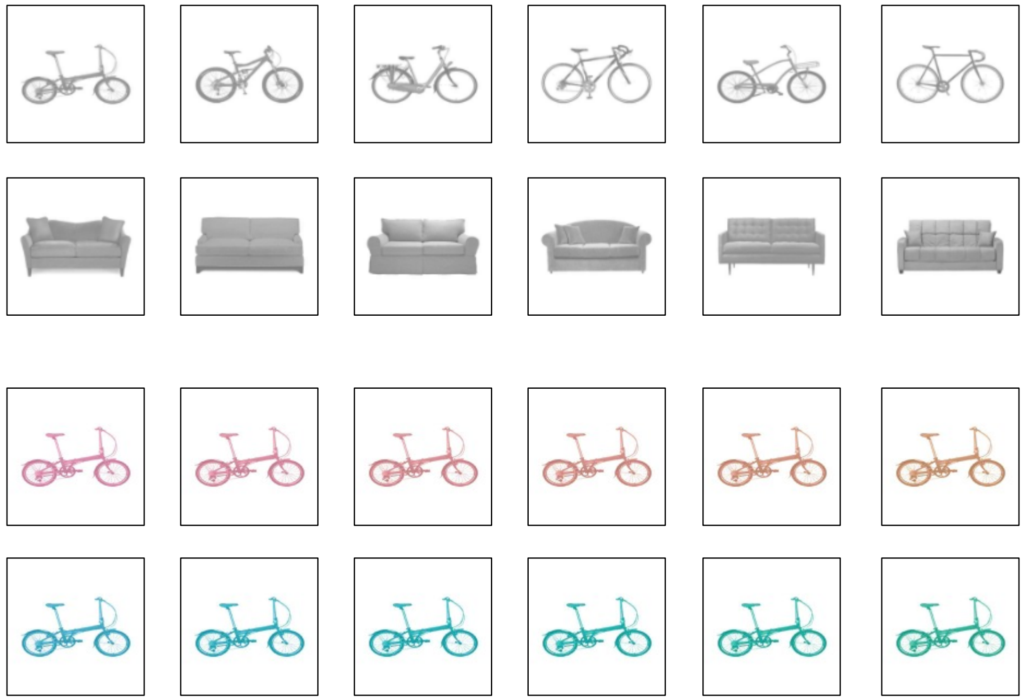

Supplementary Figure 1. Stimuli used in the experiment. **A.** The six bike and couch stimuli used. **B.** The six red and green colors used. The colors were matched in luminance and saturation and were selected from the CIELUV color space.

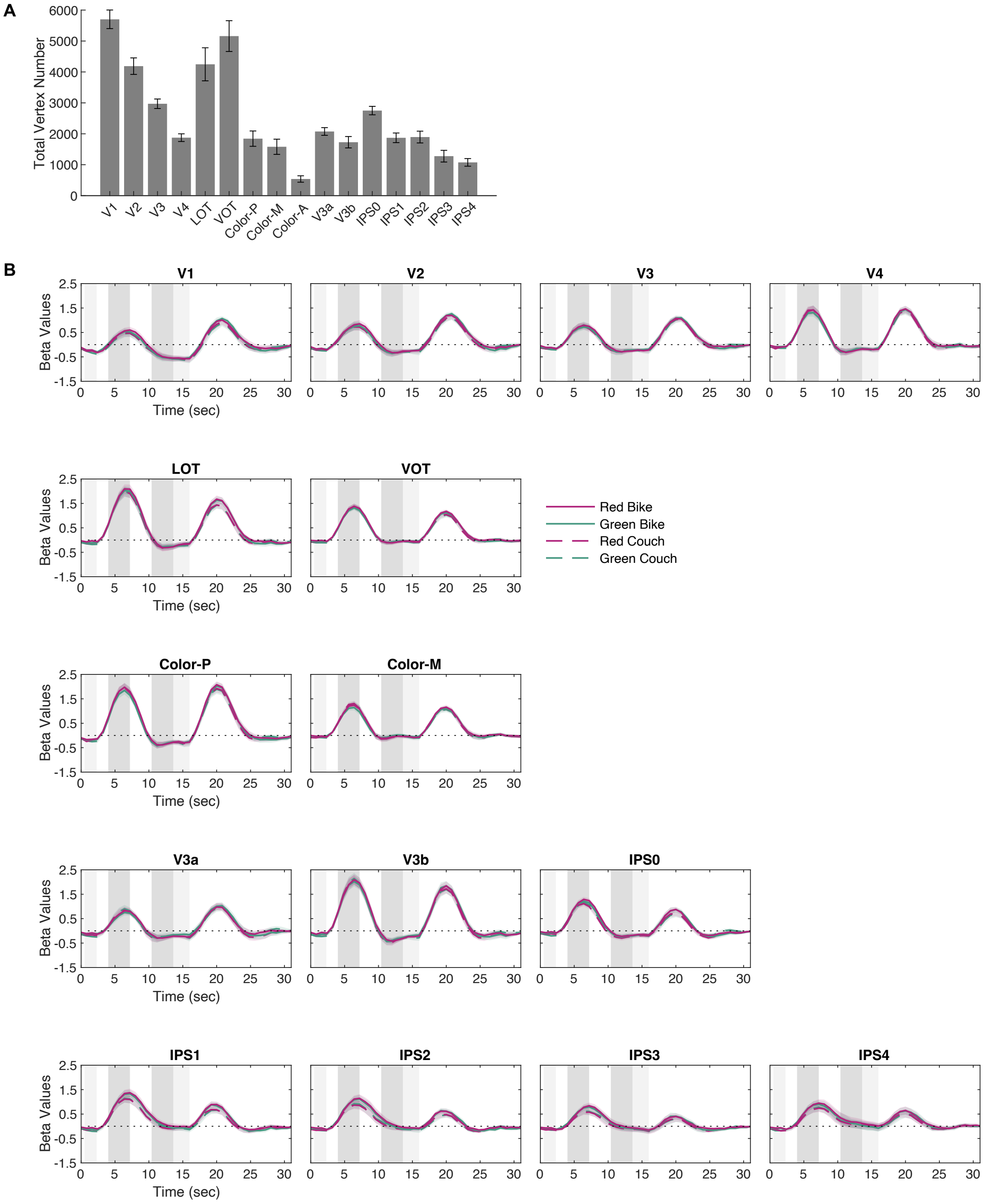

Supplementary Figure 2. Vertex numbers and response time courses of each ROI. **A.** Average number of vertices in each ROI. Error bars indicate s.e. **B.** Time courses of beta weights for the four trial conditions averaged over all the vertices within each ROI. In each ROI plot, the light gray vertical bars mark the stimulus presentation time during the encoding and probe periods, the medium gray vertical bars mark the fMRI decoding period during VWM encoding and delay. See Methods for more details. The lighter-colored ribbons around the plot lines represent s.e.

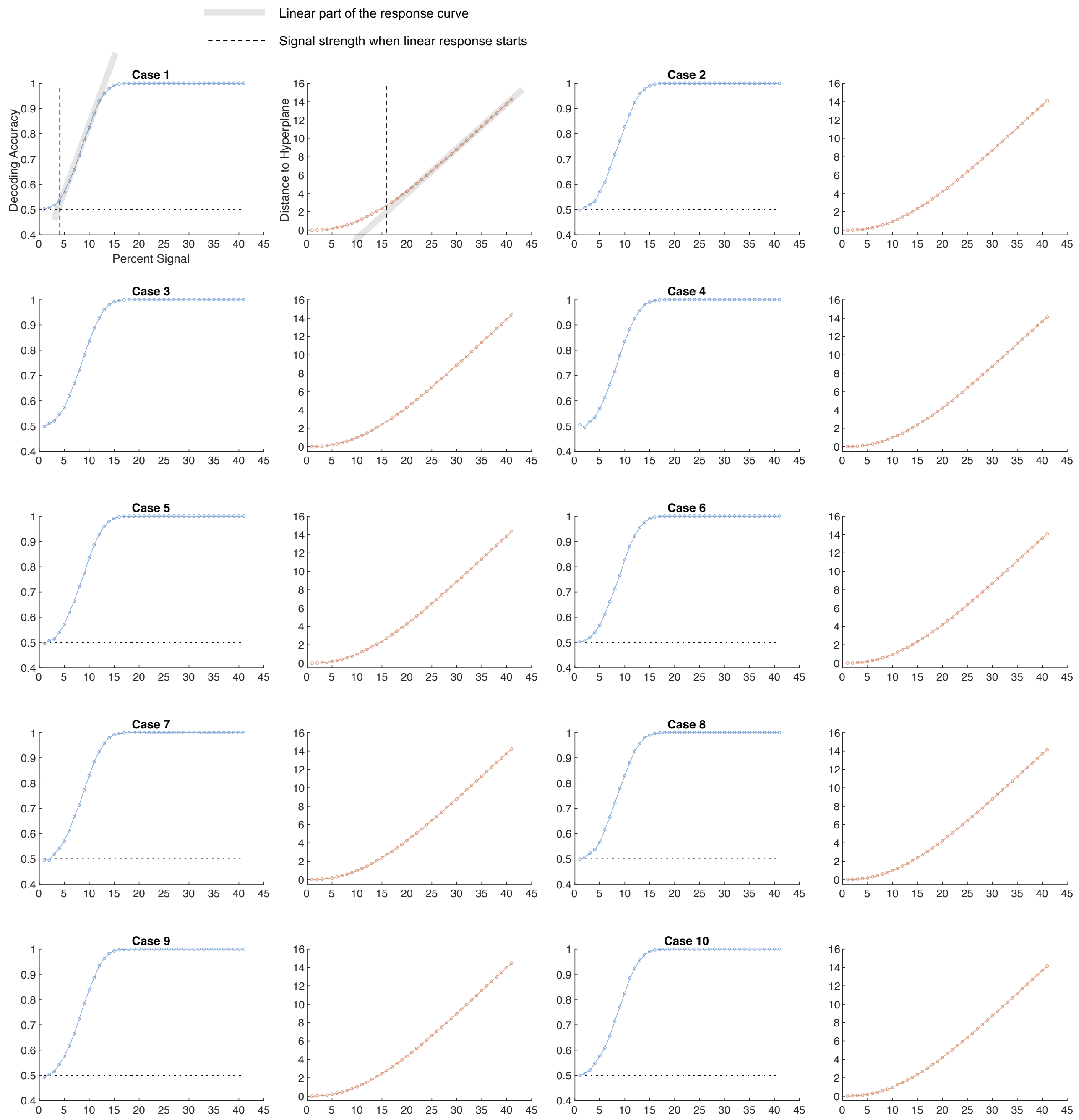

Supplementary Figure 3. Simulation results from 10 cases. Each case is the average result of 1,000 independent simulations. Blue lines depict decoding accuracy as a function of the underlying signal strength. Orange lines depict distance-to-hyperplane as a function of the underlying signal strength. The linear part of the response curve starts later with the distance-to-hyperplane than with the decoding accuracy measure. See Supplementary Results for more details.

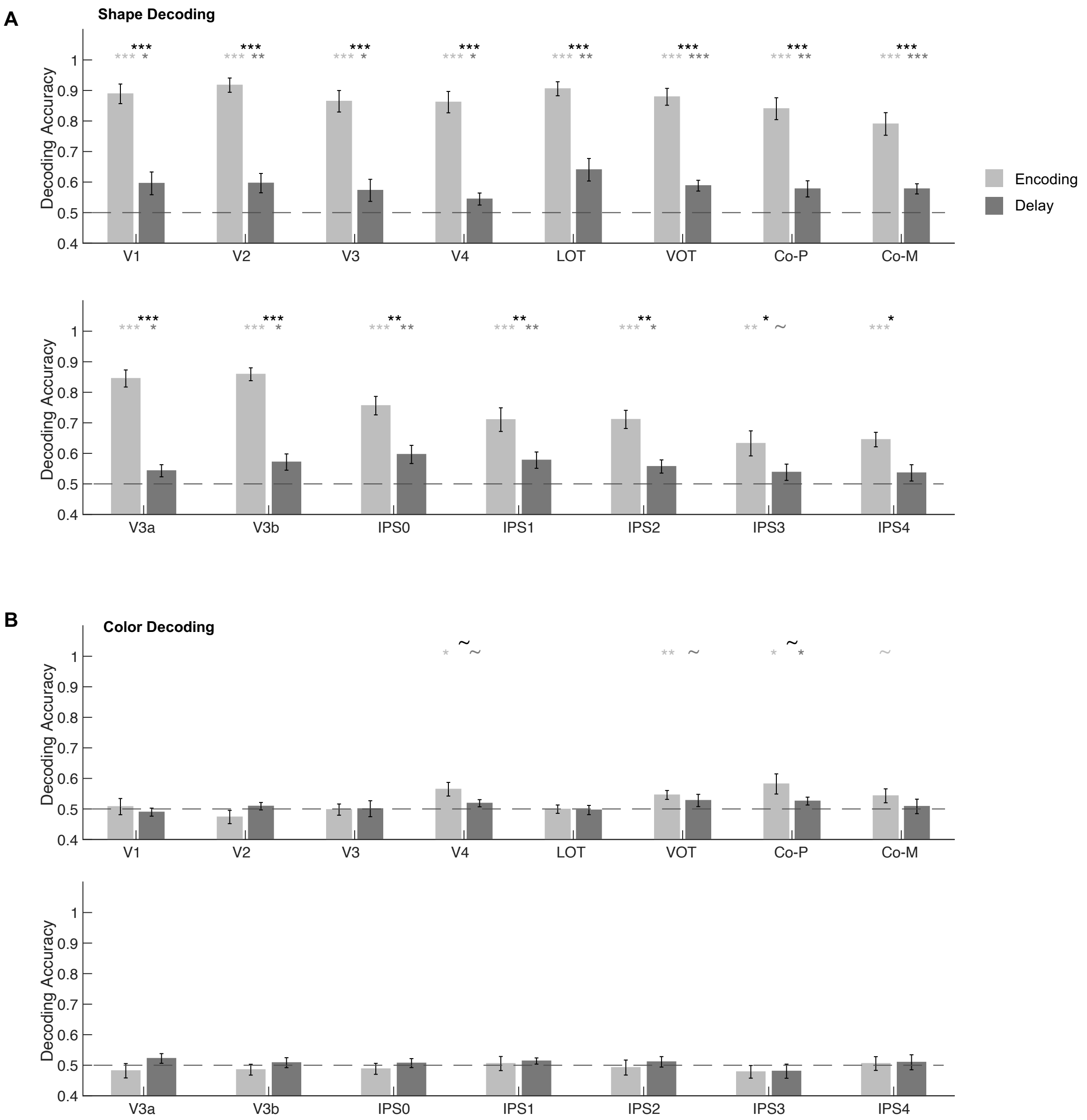

<u>Supplementary Figure 4.</u> Object shape and color feature decoding during VWM encoding and delay periods. **A.** Object shape decoding accuracy. **B.** Object color decoding accuracy. The light grey and medium grey symbols above the bars mark the decoding significance of each bar compared to chance (.5). The black symbols mark the significance of the difference between each bar pair. Error bars indicate s.e. ~ *p* < .1, * *p* < .05, ** *p* < .01, *** *p* < .001.

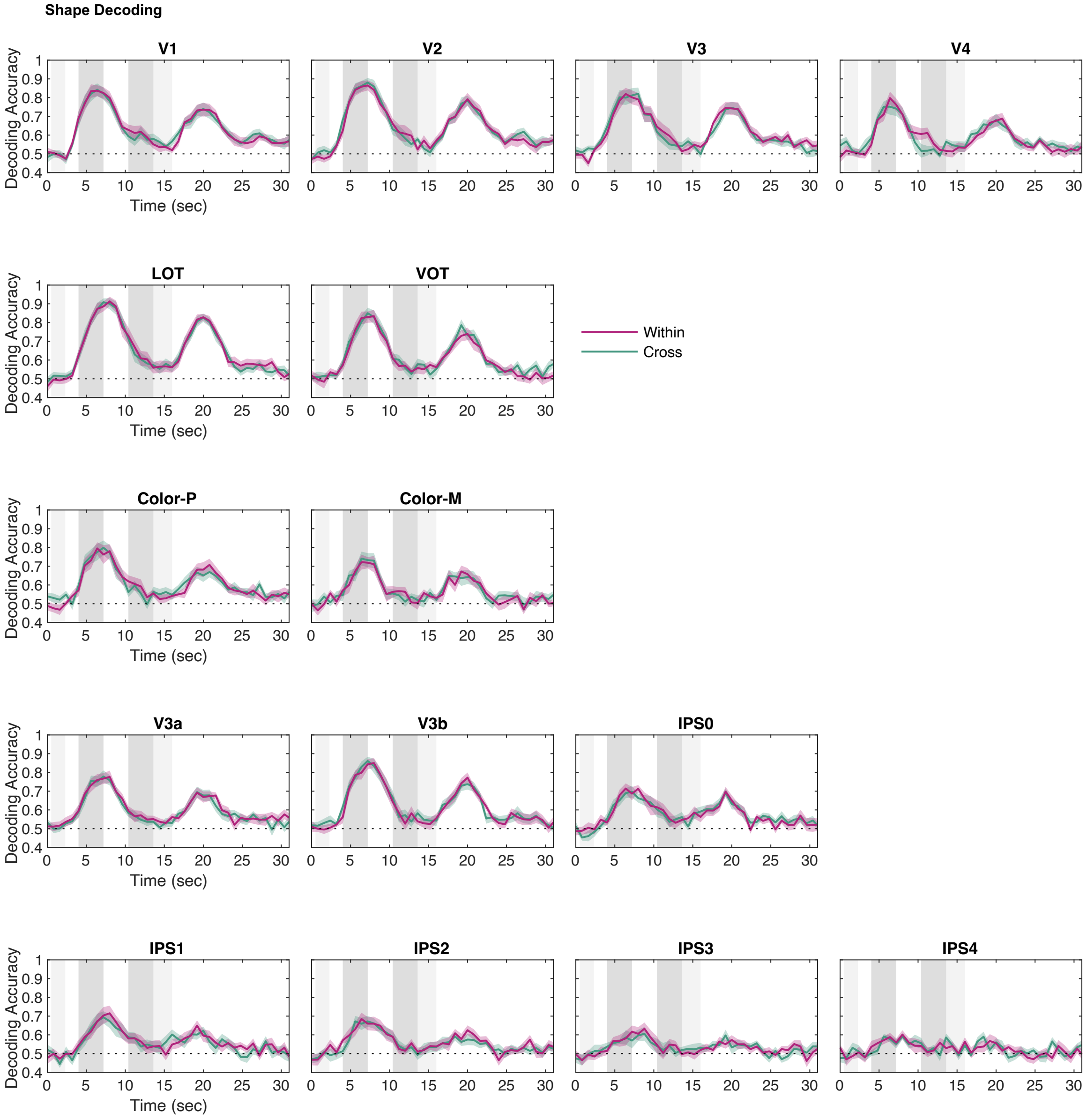

Supplementary Figure 5. Within- and cross-decoding across time. In each ROI plot, the light gray vertical bars mark the stimulus presentation time during the encoding and probe periods, the medium gray vertical bars mark the fMRI decoding period for VWM encoding and delay. See Methods for more details. The horizontal dashed line indicates chance-level decoding. The lighter-colored ribbons around the plot lines represent s.e.

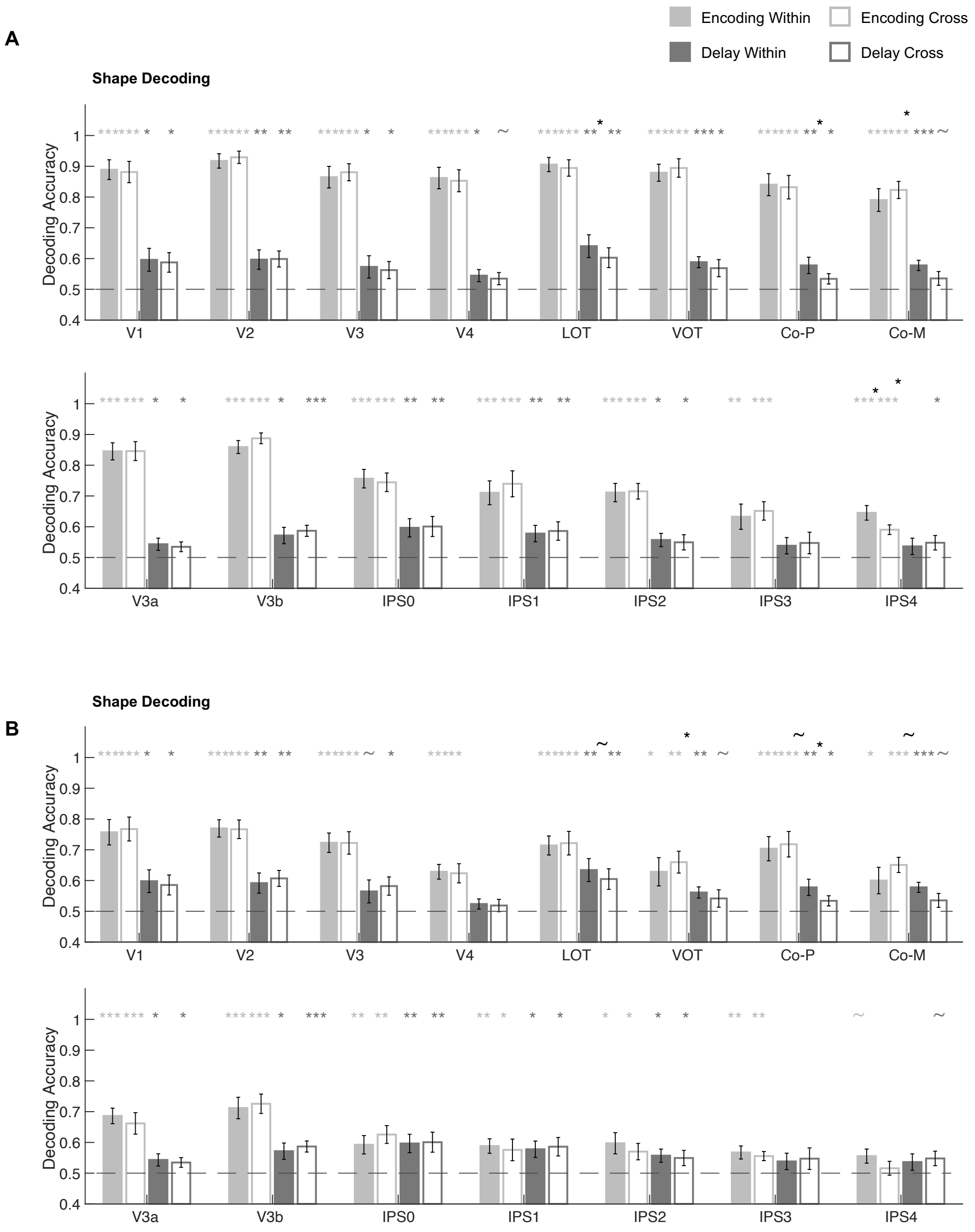

Supplementary Figure 6. Cross-color shape-decoding results for the individual ROIs. **A.** Results from the encoding period and the delay period. **B.** Results from the early encoding period and the delay period, with the overlapping color area vertices removed from all the other ROIs. The light grey and medium grey symbols right above the bars mark the significance of each bar compared to chance (.5); the lower row of black symbols mark the significance of the cross-decoding drop in each VWM stage; and the upper row of black symbols mark the significance of the difference in cross-decoding drop between encoding and delay. See Methods for more details. Error bars indicate s.e. ~ $p < .1$, * $p < .05$, ** $p < .01$, *** $p < .001$.

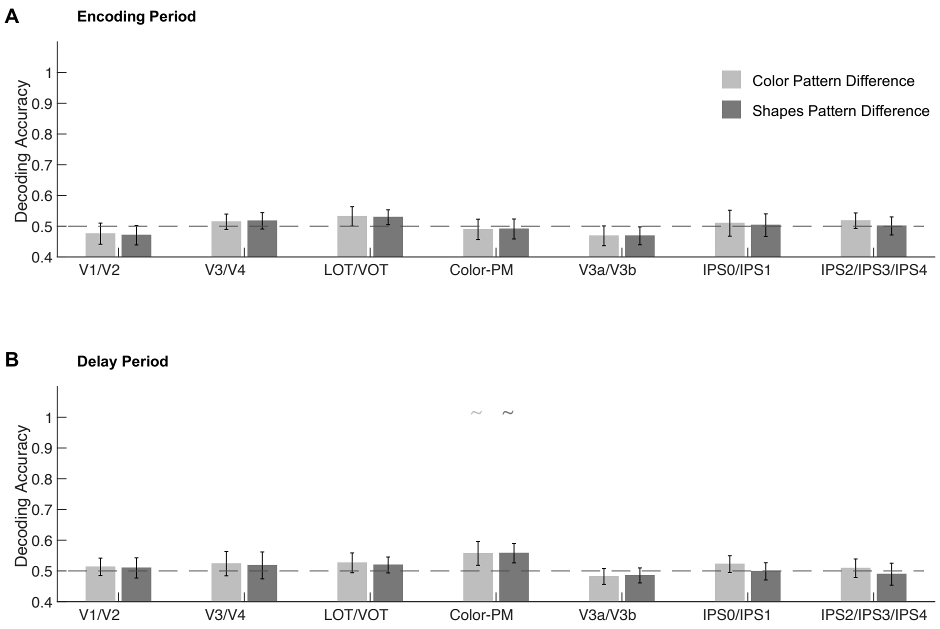

Supplementary Figure 7. Pattern difference decoding for the seven ROI pairs. **A.** Results from the encoding period. **B.** Results from the delay period. For color pattern difference decoding, we contrasted the difference between Red Bikes and Green Bikes and the difference between Red Couches and Green Couches. For shape pattern difference decoding, we contrasted the difference between Red Bikes and Red Couches and the difference between Green Bikes and Green Couches. The light grey and medium grey symbols above the bars mark the decoding significance of each bar compared to chance (.5). See Methods for more details. Error bars indicate s.e. ~ $p < .1$.
